# Effect of Epidural Dexmedetomidine as an Adjuvant to Local Anesthetics for Labor Analgesia: A Meta-Analysis of Randomized Controlled Trials

**DOI:** 10.1155/2021/4886970

**Published:** 2021-10-27

**Authors:** Nijuan Li, Li Hu, Chunping Li, Xuelin Pan, Yong Tang

**Affiliations:** Department of Anesthesia, Sichuan Jinxin Women and Children Hospital, Chengdu 610000, China

## Abstract

**Background:**

This study aims to determine the analgesic effect and safety of dexmedetomidine as an adjuvant to epidural local anesthetics during labor.

**Methods:**

Randomized controlled trials comparing epidural blocks with or without dexmedetomidine for labor analgesia were comprehensively searched. Review manager 5.4 was used to analyze the extracted data.

**Results:**

Compared with placebo and opioids, dexmedetomidine relieved labor pain of 15 min (*P*=0.002), 30 min (*P*=0.01), and 120 min (*P*=0.02) after block and at the moment of fetal disengagement (*P*=0.0002), decreased mean arterial pressure of 120 min (*P*=0.01), heart rate of 30 min (*P*=0.003), 60 min (*P* < 0.00001), and 120 min (*P* < 0.00001) after block, blood loss (*P*=0.02), and the incidence of nausea/vomiting (*P*=0.006), and increased the incidence of maternal bradycardia (*P*=0.04). However, sensitivity analysis only found that the incidence of nausea/vomiting was significantly different. Compared with placebo, dexmedetomidine relieved labor pain of 30 min after block (*P* < 0.00001) and did not increase the incidences of side effects, but only two studies were enrolled. Compared with opioids, dexmedetomidine decreased the incidence of nausea/vomiting (*P*=0.002), increased the incidence of maternal bradycardia (*P*=0.04), and had a similar effect on labor pain relief; however, sensitivity analysis found that significant difference existed only at the incidence of nausea/vomiting. Other outcomes from meta-analysis or subgroup analysis were not different.

**Conclusions:**

Epidural dexmedetomidine has the potential to offer a better analgesic effect than placebo, similar labor pain control to opioids, and has no definite adverse effects on the parturient or fetus, but more high-quality studies are needed to confirm these conclusions.

## 1. Introduction

Labor pain causes significant suffering to parturients, both physically and mentally. Additionally, it increases the risk of childbirth [1, 2] and, to some extent, increases the rate of cesarean section [3]. Labor analgesia refers to the use of various methods to reduce or eliminate maternal labor pain and is increasingly applied in obstetrics [3]. The features of ideal labor analgesia include maternal and fetal safety, rapid onset, good analgesic effect, and few adverse reactions [3].

Currently, epidural analgesia with local anesthetics has been the most effective and preferred choice for labor and delivery [4, 5]. However, it also has some disadvantages during labor, including motor blockade, maternal hypotension, longer second stage of labor, and urinary retention [6]. Thus, a major challenge for anesthesiologists is the correct balance of local anesthetics and the management of complications. Local anesthetics combined with opioids were used to address these challenges. However, it has been shown that epidural opioids are often accompanied by adverse effects, such as respiratory depression, lethargy, pruritus, nausea, and vomiting [7].

Dexmedetomidine, a highly selective *α*2-adrenoceptor agonist, possesses anxiolytic, sedative, and analgesic properties without causing respiratory depression [8, 9]. Moreover, dexmedetomidine combined with local anesthetics has been successfully used for epidural labor analgesia with few side effects [10, 11]; however, no published meta-analysis has evaluated the effect of epidural dexmedetomidine on labor analgesia. To provide a high evidence level for clinical application, we conducted a meta-analysis comparing dexmedetomidine with placebo or opioids as an adjuvant to local anesthetics for labor analgesia with respect to analgesic effect and safety.

## 2. Methods

This meta-analysis was conducted by following recommendations of the Cochrane Handbook for Systematic Reviews of Interventions [12] and the Preferred Reporting Items for Systematic Reviews and Meta-Analyses (PRISMA) [13]. The study was registered with PROSPERO (CRD42020167287).

### 2.1. Search Strategy

Two authors comprehensively searched Embase (1980–2020.08), PubMed (1966–2020.08), Medline (1966–2020.08), and the Cochrane Library using keywords (“labor OR labour OR vaginal delivery OR vaginal,” “analgesia OR pain,” and “dexmedetomidine”) without language restriction. The databases of https://clinicaltrials.gov/ and Chinese Clinical Trial Registry were also searched by using the following keywords: “labor OR labour OR vaginal delivery OR vaginal” and “dexmedetomidine.” The search strategy can be found in Appendix A. The references of the identified trials and systematic reviews were also manually searched to identify any potentially relevant trials.

### 2.2. Inclusion Criteria

Trials were included in our meta-analysis to determine whether they met the PICOS (patients, intervention, comparator, outcome, and study design) criteria. (1) Patients: nulliparous women undergoing epidural anesthesia during labor. (2) Intervention: epidural block with epidural dexmedetomidine for labor analgesia without dosage restriction. (3) Comparator: epidural block with or without epidural opioids (morphine, fentanyl, sufentanil, and remifentanil) for labor analgesia. (4) Outcomes: visual analog scale (VAS) for pain, mode of delivery, duration of labor, blood pressure, heart rate (HR), blood loss, onset of analgesia, motor block, level of sedation, complications, fetal 1-min and 5-min Apgar scores, and umbilical artery pH and partial pressure of oxygen (PaO_2_). (5) Study design: randomized controlled trials (RCTs). Trials with insufficient outcome data were excluded. Two authors independently assessed the eligibility of studies. In cases of disagreement, a consensus was reached through discussion with a third author where necessary.

### 2.3. Data Extraction

Two reviewers independently retrieved the relevant data from the articles using a standard data extraction form. The primary outcome was the VAS score on a 0–10 scale during labor. If the pain scale was assessed by a 0–100 scale, then, it was converted to a 0–10 scale by dividing by 10. The secondary outcomes were the mode of delivery, duration of labor, onset of analgesia, blood pressure, HR, blood loss, motor block, level of sedation, complications, 1-min and 5-min Apgar scores, and umbilical artery pH and PaO_2_. Missing data were requested from study authors.

### 2.4. Assessment of Methodological Quality

Two authors assessed the quality of the included studies independently based on the guidelines in the Cochrane Handbook for Systematic Reviews of Interventions and a modified Jadad 7-point scale. A study with a modified Jadad score <4 points is regarded as low-quality [14]. If the number of included RCTs was ≥10, publication bias was assessed by a funnel plot [15].

### 2.5. Statistical Analysis

Review Manager software 5.4 was used for meta-analysis. Mean differences (MDs) with 95% confidence intervals (CIs) were used to assess continuous outcomes. Odds ratios (ORs) with 95% CIs were used to assess dichotomous outcomes. Statistical heterogeneity among the included studies was assessed by *P* and *I*^2^. The control group included placebo and opioids, so all meta-analyses were conducted by using a random-effect model. The inverse variance and Mantel–Haenszel methods were used to combine separate statistics. Sensitivity analysis was conducted by omitting one study in turn to examine the reliability and conclusiveness of the available evidence. Subgroup analysis was conducted in accordance with different controls (placebo or opioids). A *P* value less than 0.05 was considered significant.

Additionally, trial sequential analysis (TSA) software 0.9.5.10 Beta was used to examine the reliability and conclusiveness of the available evidence according to a previous meta-analysis [16, 17]. To calculate the required information size (RIS), all outcomes used two-sided tests with a type I error of 5% and a power of 80%, continuous outcomes used an empirical mean difference, and dichotomous outcomes used a low bias-based relative risk reduction and a literature-based incidence in controls (5% for nausea/vomiting [18]).

## 3. Results

### 3.1. Search Results

**Figure 1:**
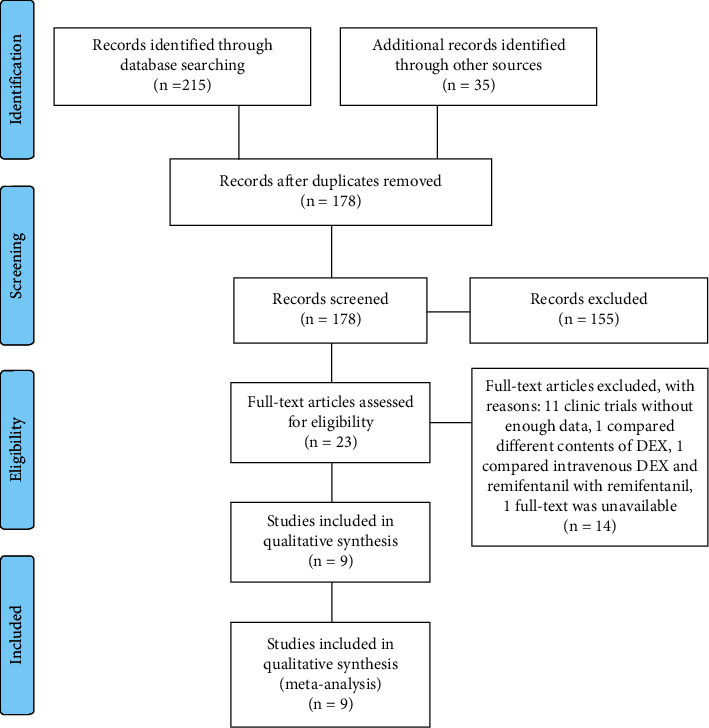
PRISMA flow chart for study selection.

Throughout the search strategy, a total of 178 studies were identified, and 155 studies were excluded by reading the title and abstract (Figure 1). Then, 11 registered clinical trials with no data available and one study [19] with no full text available were excluded. Two further articles were excluded after reading the full texts of the remaining article in detail. One article [10] compared different contents of dexmedetomidine, and the second [20] compared intravenous dexmedetomidine and remifentanil with remifentanil. Finally, nine studies [3, 11, 21–27] with 1,403 patients were included in our meta-analysis.

The basic characteristics and interventions are summarized in Table 1. Eight RCTs assessing ropivacaine were carried out in China [3, 11, 22–27], and one assessing bupivacaine was carried out in Egypt [21]. Five studies [3, 11, 22, 25, 27] compared dexmedetomidine with placebo, one [21] compared dexmedetomidine with fentanyl, and three [23, 24, 26] compared dexmedetomidine with sufentanil. Six RCTs [3, 22–26] used 0.5 *μ*g/mL dexmedetomidine, one [11] used 0.5 *μ*g/kg dexmedetomidine, one [21] used 1 *μ*g/kg dexmedetomidine, and one [27] used four concentrations of dexmedetomidine. Eight studies [3, 11, 21, 23–27] with Jadad scores ≥4 were regarded as of high-quality.

### 3.2. Risk of Bias Assessment

**Figure 2:**
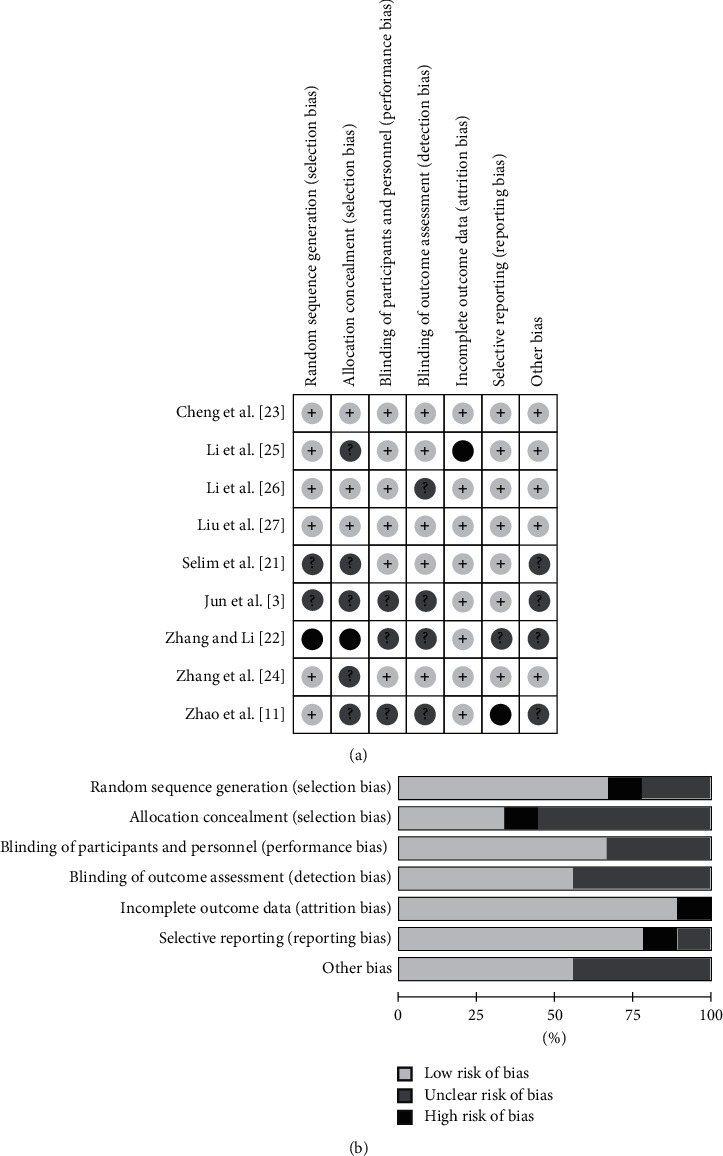
Risk of bias summary (a) and graph (b) of the included studies.

The risk of bias assessments is presented in Figure 2. Six RCTs [11, 23–27] were recorded using a computer or a random number generator for randomization, and three [23, 26, 27] reported allocation concealment via sealed envelopes. A double-blind method was carried out in six studies [21, 23–27], and the implementation of blinding of outcomes was carried out in five studies [21, 23, 25, 27]. One study [25] had a high risk of bias for incomplete outcome data, and one study [11] had a high risk of bias for selective reporting. Five studies [23–27] presented no other bias.

### 3.3. Results of Meta-Analysis

#### 3.3.1. Primary Outcomes

Statistical differences in VAS scores (Table 2, Supplementary Figure 1) between the dexmedetomidine group and control group were detected at 15 min (*P*=0.002), 30 min (*P*=0.01), and 120 min (*P*=0.02) after block and at the moment of fetal disengagement (*P*=0.0002). However, heterogeneities existed among the included studies (except VAS score before epidural block, others had *I*^2^ >50%, Table 2 and Supplementary Figure 1).

#### 3.3.2. Secondary Outcomes

Statistical differences (Table 2, Supplementary Figures 2–5) between the dexmedetomidine group and control group were detected with the measurement of mean arterial pressure (MAP) of 120 min (*P*=0.01) after block, HR of 30 min (*P*=0.003), 60 min (*P* < 0.00001), and 120 min (*P* < 0.00001) after block, blood loss (*P*=0.02), and incidences of maternal bradycardia (*P*=0.04) and nausea/vomiting (*P*=0.006). Other outcomes, including the duration of first and second stages, mode of delivery, onset of analgesia, MAP of 15 min, 30 min, and 60 min after block and at the moment of fetal disengagement, HR of 15 min after block and the moment of fetal disengagement, level of motor block assessed by a modified Bromage scale, 1-min and 5-min Apgar scores, umbilical artery pH and PaO_2_, and incidences of hypotension, itching, shivering, urinary retention, and fetal HR abnormality, were not significantly different (Table 2, Supplementary Figures 2–11). Heterogeneities among studies were detected at the time of the first (*I*^2^ = 92%) and second (*I*^2^ = 97%) stages, onset of analgesia (*I*^2^ = 93%), MAP of 15 min (*I*^2^ = 91%) and 30 min (*I*^2^ = 52%) after block, and at the moment of fetal disengagement (*I*^2^ = 92%), HR of 15-min after block (*I*^2^ = 93%) and at the moment of fetal disengagement (*I*^2^ = 96%), level of motor block (*I*^2^ = 95%), and umbilical artery PaO_2_ (*I*^2^ = 96%).

### 3.4. Sensitivity Analysis and Subgroup Analysis and TSA

To examine the reliability and conclusiveness of the positive results mentioned above, sensitivity analysis was conducted and we found that the incidence of nausea/vomiting was significantly different regardless of which study was omitted, and no heterogeneities were detected (data not shown). To further confirm the conclusiveness of the incidence of nausea/vomiting, TSA was performed, and the *Z* curve crossed the conventional boundary, TSA boundary, and RIS (Supplementary Figure 12).

Subgroup analysis of all outcomes was conducted through different controls (placebo or opioids). Compared with placebo (Table 3), a significant difference was detected at a VAS score of 30 min after block (*P* < 0.00001), but sensitivity analysis could not be conducted because only two studies of 158 participants were enrolled. Compared with opioids (Table 3), significant differences were detected regarding the incidences of maternal bradycardia (*P*=0.04, *I*^2^ = 0%) and nausea/vomiting (*P*=0.002, *I*^2^ = 0%) with no heterogeneities. To confirm the reliability and conclusiveness of the results of the subgroup analysis, sensitivity analysis was further carried out, and it was only found that the incidence of nausea/vomiting always had significant differences regardless of which study was omitted (data not shown).

## 4. Discussion

This meta-analysis was performed to investigate the effectiveness and safety of epidural dexmedetomidine during labor analgesia. In total, five RCTs comparing dexmedetomidine with placebo and four RCTs comparing dexmedetomidine with opioids were enrolled for meta-analysis. Pooled data from our meta-analysis found that compared with placebo and opioids, epidural dexmedetomidine decreased the VAS score, MAP, HR, blood loss, and the incidence of nausea/vomiting, and increased the incidence of maternal bradycardia. However, sensitivity analysis only found that the incidence of nausea/vomiting always had significant differences. Moreover, when compared with placebo, subgroup analysis found that dexmedetomidine relieved labor pain and did not increase the incidences of side effects; however, sensitivity analysis of labor pain could not be conducted for only two studies enrolled. When compared with opioids, subgroup analysis found that dexmedetomidine decreased the incidence of nausea/vomiting, increased the incidence of maternal bradycardia, and had similar effect on labor pain. However, sensitivity analysis only found that the incidence of nausea/vomiting always had significant differences. In addition, both meta-analysis and subgroup analysis found no significant differences in other outcomes. Taken together, this meta-analysis found that epidural dexmedetomidine had the potential to alleviate labor pain and did not increase the incidences of adverse effects compared with placebo, provided similar analgesic effects and decreased the incidence of nausea/vomiting compared with opioids, indicating that dexmedetomidine can replace opioids and be safely used in epidural block during labor analgesia.

Although many meta-analyses have demonstrated that single-shot injection of subarachnoid or epidural dexmedetomidine prolongs the duration of analgesia and decreases the requirement for rescue analgesia compared with placebo [28–32], opioids [33], or clonidine [34] in different surgical procedures, including cesarean section, no meta-analysis evaluated the effect of epidural dexmedetomidine on labor analgesia. In this meta-analysis, when compared with the control (placebo and opioids), epidural dexmedetomidine relieved labor pain of 30 min after block and the moment of fetal disengagement. However, this difference was unstable by sensitivity analysis. When compared with placebo, dexmedetomidine relieved labor pain of 30 min after block, but only two studies were enrolled; when compared with opioids, dexmedetomidine showed a similar effect of pain control. Taken together, these results indicate that dexmedetomidine as an adjuvant to local anesthetics may offer better pain control during labor than placebo, but more high-quality studies are needed to strengthen this conclusion.

The greatest concern of epidural dexmedetomidine administration is safety. According to these meta-analyses [28, 31], although epidural dexmedetomidine significantly reduced the incidence of bradycardia compared with placebo, the incidence of hypotension was not different, and the overall risk of hypotension and bradycardia was statistically insignificant. Similarly, no definite conclusions were reached for epidural dexmedetomidine to increase the incidences of hypotension and bradycardia when compared with placebo and/or opioids in this study. Nausea and vomiting are the most common side effects of epidural anesthesia [28]. Three prior meta-analyses [28, 31, 32] found that the incidence of nausea/vomiting was not different between epidural dexmedetomidine and placebo. A recent meta-analysis [35] has found that epidural dexmedetomidine significantly reduced the incidence of nausea/vomiting compared with epidural opioids, which is completely consistent with our results. Taken together, epidural dexmedetomidine has no definite adverse effects on the parturient.

Different from other surgeries, the safety of the fetus should be considered in the cesarean section and labor analgesia. Compared with placebo, spinal anesthesia with intrathecal dexmedetomidine had no significant differences in the 1-min or 5-min Apgar scores or umbilical arterial pH and PaO_2_ [29]. In this study, epidural dexmedetomidine had no differences in the duration of stages, mode of delivery, 1-min or 5-min Apgar scores, umbilical artery pH and PaO_2_, and fetal HR with placebo and/or opioids, indicating that epidural labor analgesia with dexmedetomidine is safe for fetuses.

Some limitations are present in our meta-analysis. Except for the incidence of nausea/vomiting, due to the small sample size and heterogeneity among the included studies, other outcomes were unable to draw firm conclusions; additional high-quality RCTs are required to fully evaluate these outcomes. The actual dose of dexmedetomidine was not mentioned in all enrolled RCTs; thus, this study did not provide any insight into the effect of different doses of dexmedetomidine. Moreover, eight of the included studies were from China, which resulted in geographical limitations of this study.

## 5. Conclusion

Dexmedetomidine as an adjuvant to epidural local anesthetics has the potential to offer a better analgesic effect than placebo, similar labor pain control to opioids, and has no definite adverse effects on the parturient or fetus. Still, more high-quality RCTs are needed to confirm these conclusions.

## Figures and Tables

**Table 1:** The characteristics of included studies.

Study (year)	Country	DEX/control							Epidural analgesia	DEX intervention	Control intervention	Jadad score
		Cases	Mean age (years)	Gestational week	Weight (kg)	Height (cm)	BMI (kg/m^2^)	Cervical dilatation (cm)				
Jun et al. [3]	China	75/75	25.5/26.5	37.6/36.9	61.4/62.8	166.1/165.5	NM	NM	When the cervical dilation reached 3 cm, a catheter was inserted at the L3-L4 interspace, 3–4 cm cephaladly. First dose: 10 mL, background: 10 mL/h, bonus: 5 mL, lockout time: 20 min	PCEA: 0.1% ropivacaine and 0.5 *μ*g/mL DEX; first dose: 10 ml, background infusion: 10 ml/h, a single additional quantity: 5 ml, and lock time: 20 min	PCEA: 0.1% ropivacaine; first dose: 10 ml, background infusion: 10 ml/h, a single additional: quantity 5 ml, and lock time: 20 min	4

Zhao et al. [11]	China	40/40	25.9/26.2	NM	80.7/79.3	163.2/163.03	30.3/29.9	NM	A catheter was advanced through the needle 4 cm into the epidural space	0.125% ropivacaine with 0.5 *μ*g/kg DEX	0.125% ropivacaine	5

Selim et al. [21]	Egypt	44/43	25.1/24.0	38.9/39.2	74.7/78.1	161/163	NM	5.1/4.9	A catheter was inserted into the L3-L4 interspace, 2–3 cm cephaladly. Initial bolus 17 mL, a second dose was injected when VAS was ≥4	12 mL of 0.25% bupivacaine plus 1 *μ*g/kg DEX diluted in 5 mL saline	12 mL of 0.25% bupivacaine plus 1 *μ*g/kg fentanyl diluted in 5 mL saline	5

Zhang and Li [22]	China	30/30	28.0/26.9	39.2/38.7	71.3/68.5	159.4/160.5	NM	2.0/2.1	When the cervical dilation reached 2 cm, a catheter was inserted at the L2-L3 interspace, 3–5 cm cephaladly. First dose 10 mL, background 8 mL/h, bonus 8 mL, lockout time 15 min	10 ml of 0.1% ropivacaine plus 0.5 *μ*g/mL DEX, background infusion 8 ml/h, a bolus of 8 ml when VSA > 7, lockout time 15 min	10 ml of 0.1% ropivacaine, background infusion: 8 ml/h, a bolus of 8 ml when VSA > 7, lockout time: 15 min	3

Cheng et al. [23]	China	80/80	27.5/27.4	39.4/39.4	NM	NM	25.9/26.3	NM	When the cervical dilation reached 3 cm, a catheter was inserted at the L3-L4 interspace, 3–4 cm cephaladly. Initial bolus 13 mL, background 8 mL/h, lockout interval 30 min	PCEA: 0.125% or 0.08% ropivacaine with 0.5 *μ*g/mL DEX; first dose: 10 ml, background infusion: 8 ml/h, a single additional quantity: 8 ml, and lock time: 30 min	PCEA: 0.125% or 0.08% ropivacaine with 0.5 *μ*g/mL sufentanil; first dose: 10 ml, background infusion: 8 ml/h, a single additional quantity: 8 ml, and lock time 30 min	7

Zhang et al. [24]	China	36/34	27.3/26.7	39.8/40.1	70.8/69.5	159.3/160.4	NM	NM	When the cervical dilation reached 2 cm, a catheter was inserted at the L2-L3 interspace, 3–4 cm cephaladly. First dose: 10 mL, background: 6 mL/h, bonus: 6 mL, lockout time: 20 min	PCEA: 0.1% ropivacaine plus 0.5 *μ*g/mL DEX; first dose: 10 ml, background infusion: 6 ml/h, a single additional quantity: 6 ml, and lock time: 20 min	PCEA: 0.1% ropivacaine plus 0.5 *μ*g/mL sufentanil; first dose: 10 ml, background infusion: 6 ml/h, a single additional quantity: 6 ml, and lock time: 20 min	6

Li et al. [25]	China	291/287	28.8/29.1	39.2/39.4	69.4/68.8	160.2/160.6	NM	NM	When the cervical dilation reached 2 cm, a catheter was inserted at the L2-L3 interspace, 3–5 cm cephaladly. First dose: 10 mL, background: 6 ml/h, bolus: 6 ml, lockout time: 15 min	PCEA: 0.1% ropivacaine with 0.5 *μ*g/mL DEX; first dose: 10 ml, background infusion: 6 ml/h, a single additional quantity: 6 ml, and lock time: 15 min	PCEA: 0.1% ropivacaine; first dose: 10 ml, background infusion: 6 ml/h, a single additional quantity: 6 ml, and lock time: 15 min	6

Li et al. [26]	China	36/35	29.2/30.1	39.1/37.4	65.2/64.7	159.6/159.0	NM	NM	When the cervical dilation reached 3 cm, a catheter was inserted at the L2-L3 interspace, 3–4 cm cephaladly. First dose: 10 mL, background: 7 ml/h, bolus: 7 ml, lockout time: 25 min	PCEA: 0.1% ropivacaine with 0.5 *μ*g/mL DEX; first dose: 10 ml, background infusion: 7 ml/h, a single additional quantity: 7 ml, and lock time: 25 min	PCEA: 0.1% ropivacaine with 0.5 *μ*g/ml sufentanil; first dose: 10 ml, background infusion: 7 ml/h, a single additional quantity: 7 ml, and lock time: 25 min	7

Liu et al. [27]	China	118/29	27.3/27	39.3/40	69.5/70	160.5/160	NM	3/3	A catheter was inserted at the L3-L4 interspace, 3–4 cm cephaladly. First dose: 13 mL	13 ml Ropivacaine with 0.3, 0.4, 0.5, or 0.6 *μ*g/mL DEX	13 ml ropivacaine	7

DEX, dexmedetomidine; BMI, body mass index; NM, not mentioned; VAS, visual analog scale.

**Table 2:** Results of meta-analysis.

Outcomes	Studies	Participants	Effect estimate		
			MD (OR) and 95% CI	*I* ^2^ (%)	*P*
VAS score (before block)	5	548	−0.02 [−0.19, 0.16]	15	0.85
VAS score (15 min after block)	2	240	−2.15 [−3.53, −0.77]	92	0.002
VAS score (30 min after block)	4	388	−0.97 [−1.75, −0.19]	93	0.01
VAS score (60 min after block)	3	238	−1.03 [−2.17, 0.11]	92	0.08
VAS score (120 min after block)	3	232	−1.05 [−1.90, −0.20]	86	0.02
VAS score (at the moment of disengagement)	3	379	−0.96 [−1.46, −0.46]	94	0.0002
Time of first stage	8	1305	−18.51 [−37.26, 0.25]	92	0.05
Time of second stage	8	1300	1.28 [−4.72, 7.28]	97	0.68
Mode of delivery	7	674	1.13 [0.70, 1.81]	0	0.62
Onset of analgesia	4	288	0.04 [−2.51, 2.58]	93	0.98
MAP (before block)	4	477	0.68 [−1.08, 2.43]	0	0.45
MAP (15 min after block)	3	327	−0.93 [−8.32, 6.46]	91	0.81
MAP (30 min after block)	3	317	−0.92 [−3.78, 1.93]	52	0.53
MAP (60 min after block)	2	167	−3.05 [−6.16, 0.05]	39	0.05
MAP (120 min after block)	2	161	−3.74 [−6.75, −0.74]	31	0.01
MAP (at the moment of disengagement)	2	229	0.32 [−8.83, 9.47]	92	0.95
HR (before the block)	4	477	−0.41 [−1.88, 1.05]	0	0.58
HR (15 min after block)	3	327	−3.33 [−10.53, 3.87]	93	0.36
HR (30 min after block)	3	317	−2.70 [−4.47, −0.92]	48	0.003
HR (60 min after block)	2	167	−5.83 [−7.78, −3.88]	0	<0.00001
HR (120 min after block)	2	161	−4.90 [−6.66, −3.13]	0	<0.00001
HR (at the moment of disengagement)	2	229	−1.21 [−12.73, 10.30]	96	0.84
Blood loss	3	275	−6.03 [−10.93, −1.13]	0	0.02
Motor block (modified Bromage scale)	7	1162	0.29 [−0.37, 0.96]	95	0.39
Apgar score (1 min)	4	437	−0.01 [−0.11, 0.10]	8	0.88
Apgar score (5 min)	5	587	−0.00 [−0.07, 0.07]	0	0.97
Umbilical artery pH	5	508	0 [−0.01, 0.02]	0	0.72
Umbilical artery PaO_2_	2	230	1.25 [−2.88, 5.37]	96	0.55
Incidence of maternal bradycardia	8	1323	2.90 [1.04, 8.11]	0	0.04
Incidence of hypotension	7	1173	1.11 [0.38, 3.20]	37	0.85
Incidence of nausea/vomiting	9	1403	0.49 [0.30, 0.82]	0	0.006
Incidence of itching	6	598	0.46 [0.12, 1.81]	21	0.27
Incidence of shivering	3	201	0.96 [0.29, 3.16]	0	0.95
Incidence of urinary retention	5	511	0.49 [0.22, 1.10]	0	0.08
Incidence of fetal heart rate abnormality	4	464	0.91 [0.40, 2.04]	0	0.82

MD, mean difference; OR, odds ratio; CI, confidence interval; VAS, visual analog scale; MAP, mean arterial pressure; HR, heart rate.

**Table 3:** Subgroup analysis based on different controls (placebo and opioid).

Outcomes	DEX vs. placebo					DEX vs. opioids				
	Studies	Participants	Effect estimate			Studies	Participants	Effect estimate		
			MD (OR) and 95% CI	*I* ^2^ (%)	*P*			MD (OR) and 95% CI	*I* ^2^ (%)	*P*
VAS score (before block)	2	230	0.05 [−0.21, 0.31]	13	0.71	3	318	−0.12 [−0.40, 0.17]	17	0.41
VAS score (15 min after block)	1	80	Not applicable			1	160	Not applicable		
VAS score (30 min after block)	2	230	−1.56 [−1.81, −1.31]	30	<0.00001	2	158	−0.20 [−0.58, 0.18]	0	0.31
VAS score (60 min after block)	1	80	Not applicable			2	158	−0.64 [−1.80, 0.52]	84	0.28
VAS score (120 min after block)	1	74	Not applicable			2	158	−0.74 [−1.65, 0.17]	79	0.11
VAS score (at the moment of disengagement)	2	219	−0.95 [−1.87, −0.02]	93	0.05	1	160	Not applicable		
Time of first stage	5	1004	−12.44 [−34.08, 9.19]	78	0.26	3	301	−27.57 [−59.46, 4.33]	96	0.09
Time of second stage	5	999	−1.44 [−6.52, 3.64]	92	0.58	3	301	5.62 [−9.92, 21.16]	99	0.48
Mode of delivery	3	287	1.16 [0.44, 3.05]	0	0.77	4	276	1.13 [0.66, 1.93]	0	0.67
Onset of analgesia	1	60	Not applicable			3	228	0.25 [−2.89, 3.39]	95	0.87
MAP (before block)	2	230	1.89 [−0.83, 4.60]	0	0.17	2	247	−0.19 [−2.5, 2.11]	0	0.87
MAP (15 min after block)	1	80	Not applicable			2	247	1.49 [−7.43, 10.41]	93	0.74
MAP (30 min after block)	2	230	−1.16 [−6.35, 4.03]	75	0.66	1	87	Not applicable		
HR (before the block)	2	230	−1.33 [−3.19, 0.54]	0	0.16	2	247	1.06 [−1.31, 3.43]	0	0.38
HR (15 min after block)	1	80	Not applicable			2	247	−0.89 [−10.80, 9.02]	95	0.86
HR (30 min after block)	2	230	−3.14 [−6.31, 0.04]	74	0.05	1	87	Not applicable		
Blood loss	2	205	−6.55 [−13.81, 0.71]	0	0.08	1	70	Not applicable		
Motor block (modified Bromage scale)	3	785	Not applicable			4	377	0.29 [−0.37, 0.96]	95	0.39
Apgar score (1 min)	2	207	0.01 [−0.08, 0.11]	0	0.79	2	230	Not applicable		
Apgar score (5 min)	3	357	0.00 [−0.07, 0.08]	0	0.89	2	230	Not applicable		
Umbilical artery pH	2	207	0.01 [−0.01, 0.03]	0	0.35	3	301	0.00 [−0.02, 0.02]	0	0.70
Umbilical artery PaO_2_	0		Not applicable			2	230	1.25 [−2.88, 5.37]	96	0.55
Incidence of maternal bradycardia	4	935	Not applicable			4	388	2.90 [1.04, 8.11]	0	0.04
Incidence of hypotension	3	785	1.59 [0.47, 5.36]	0	0.45	4	388	0.68 [0.06, 7.98]	70	0.76
Incidence of nausea/vomiting	5	1011	0.71 [0.37, 1.36]	0	0.30	4	388	0.27 [0.12, 0.61]	0	0.002
Incidence of itching	2	210	Not applicable			4	388	0.28 [0.07, 1.13]	2	0.07
Incidence of shivering	1	60	Not applicable			2	141	0.96 [0.26, 3.54]	0	0.95
Incidence of urinary retention	2	210	Not applicable			3	301	0.49 [0.20, 1.15]	0	0.10
Incidence of fetal heart rate abnormality	1	147	Not applicable			3	317	1.09 [0.44, 2.70]	0	0.86

DEX, dexmedetomidine; MD, mean difference; OR, odds ratio; CI, confidence interval; VAS, visual analog scale; MAP, mean arterial pressure; HR, heart rate.

## Data Availability

The data used to support the findings of this study are included within the article.

## Supplementary Materials

Supplementary Materials Supplementary Figure 1: forest plot diagram showing the visual analog scale (VAS) scores. Supplementary Figure 2: forest plot diagram showing the mean arterial pressure (MAP). Supplementary Figure 3: forest plot diagram showing the heart rate (HR). Supplementary Figure 4: forest plot diagram showing the blood loss. Supplementary Figure 5: forest plot diagram showing the incidences of complications. Supplementary Figure 6: forest plot diagram showing the duration of labor stages. Supplementary Figure 7: forest plot diagram showing the mode of delivery. Supplementary Figure 8: forest plot diagram showing the onset of analgesia. Supplementary Figure 9: forest plot diagram showing the level of motor block. Supplementary Figure 10: forest plot diagram showing the Apgar score. Supplementary Figure 11: forest plot diagram showing the umbilical artery pH and PaO_2_. Supplementary Figure 12: trial sequential analysis of the incidence of nausea/vomiting compared with placebo and opioids. RIS = required information size.
