# Supplementary material for: Effect of Epidural Dexmedetomidine as an Adjuvant to Local Anesthetics for Labor Analgesia: A Meta-Analysis of Randomized Controlled Trials
Source: Evid Based Complement Alternat Med. 2021 Oct 27;2021:4886970. doi: 10.1155/2021/4886970 (PMC8568549; doi:10.1155/2021/4886970)
Supplement: Supplementary Materials — Supplementary Figure 1: forest plot diagram showing the visual analog scale (VAS) scores. Supplementary Figure 2: forest plot diagram showing the mean arterial pressure (MAP). Supplementary Figure 3: forest plot diagram showing the heart rate (HR). Supplementary Figure 4: forest plot diagram showing the blood loss. Supplementary Figure 5: forest plot diagram showing the incidences of complications. Supplementary Figure 6: forest plot diagram showing the duration of labor stages. Supplementary Figure 7: forest plot diagram showing the mode of delivery. Supplementary Figure 8: forest plot diagram showing the onset of analgesia. Supplementary Figure 9: forest plot diagram showing the level of motor block. Supplementary Figure 10: forest plot diagram showing the Apgar score. Supplementary Figure 11: forest plot diagram showing the umbilical artery pH and PaO2. Supplementary Figure 12: trial sequential analysis of the incidence of nausea/vomiting compared with placebo and opioids. RIS = required information size. [file 4886970.f1.zip › 4886970.f1/Appendix A.docx]

**Appendix A**

**Embase and Cochrane search strategy**

#1 labor [ti,ab,kw] OR labour [ti,ab,kw] OR vaginal delivery [ti,ab,kw] OR vaginal [ti,ab,kw]

#2 analgesia [ti,ab,kw] OR pain [ti,ab,kw]

#3 dexmedetomidine [ti,ab,kw]

#4 #1 AND #2 AND #3

**Pubmed search strategy**

#1 labor [TIAB] OR labour [TIAB] OR vaginal delivery [TIAB] OR vaginal [TIAB]

#2 analgesia [TIAB] OR pain [TIAB]

#3 dexmedetomidine [TIAB]

#4 #1 AND #2 AND #3

**Medline search strategy**

#1 TS=(labor) OR TS=(labour) OR TS=(vaginal delivery) OR TS=(vaginal)

#2 TS=(analgesia) OR TS=(pain)

#3 TS=(dexmedetomidine)

#4 #1 AND #2 AND #3

**ClinicalTrials.gov and Chinese Clinical Trial Registry**

#1 labor [ti] OR labour [ti] OR vaginal delivery [ti] OR vaginal [ti]

#2 dexmedetomidine [ti]

#3 #1 AND #2
