# Supplementary material for: Effect of Epidural Dexmedetomidine as an Adjuvant to Local Anesthetics for Labor Analgesia: A Meta-Analysis of Randomized Controlled Trials
Source: Evid Based Complement Alternat Med. 2021 Oct 27;2021:4886970. doi: 10.1155/2021/4886970 (PMC8568549; doi:10.1155/2021/4886970)
Supplement: Supplementary Materials — Supplementary Figure 1: forest plot diagram showing the visual analog scale (VAS) scores. Supplementary Figure 2: forest plot diagram showing the mean arterial pressure (MAP). Supplementary Figure 3: forest plot diagram showing the heart rate (HR). Supplementary Figure 4: forest plot diagram showing the blood loss. Supplementary Figure 5: forest plot diagram showing the incidences of complications. Supplementary Figure 6: forest plot diagram showing the duration of labor stages. Supplementary Figure 7: forest plot diagram showing the mode of delivery. Supplementary Figure 8: forest plot diagram showing the onset of analgesia. Supplementary Figure 9: forest plot diagram showing the level of motor block. Supplementary Figure 10: forest plot diagram showing the Apgar score. Supplementary Figure 11: forest plot diagram showing the umbilical artery pH and PaO2. Supplementary Figure 12: trial sequential analysis of the incidence of nausea/vomiting compared with placebo and opioids. RIS = required information size. [file 4886970.f1.zip › 4886970.f1/Supplementary figure.docx]

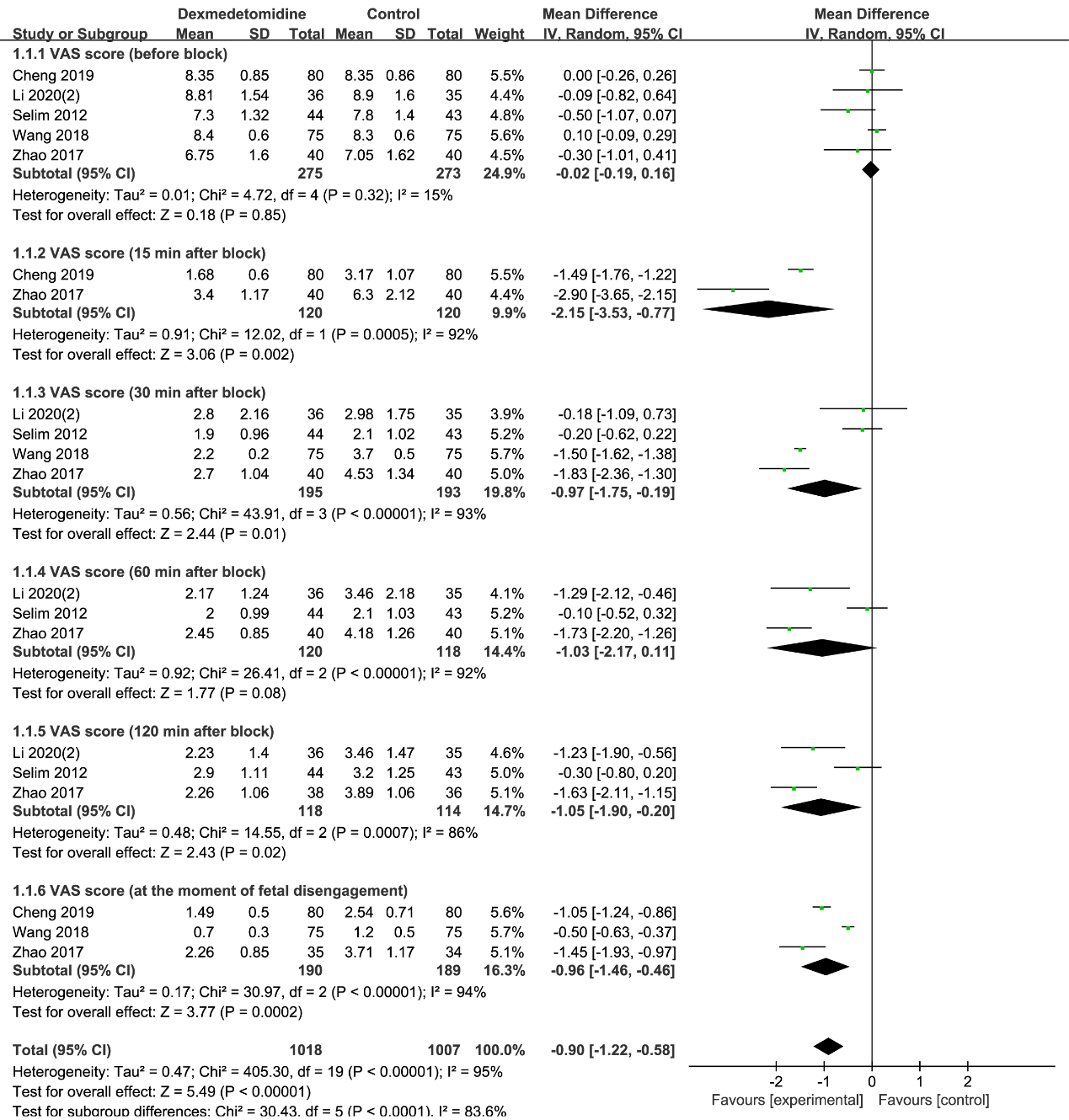


Supplementary figure. 1. Forest plot diagram showing the visual analog scale (VAS) scores.


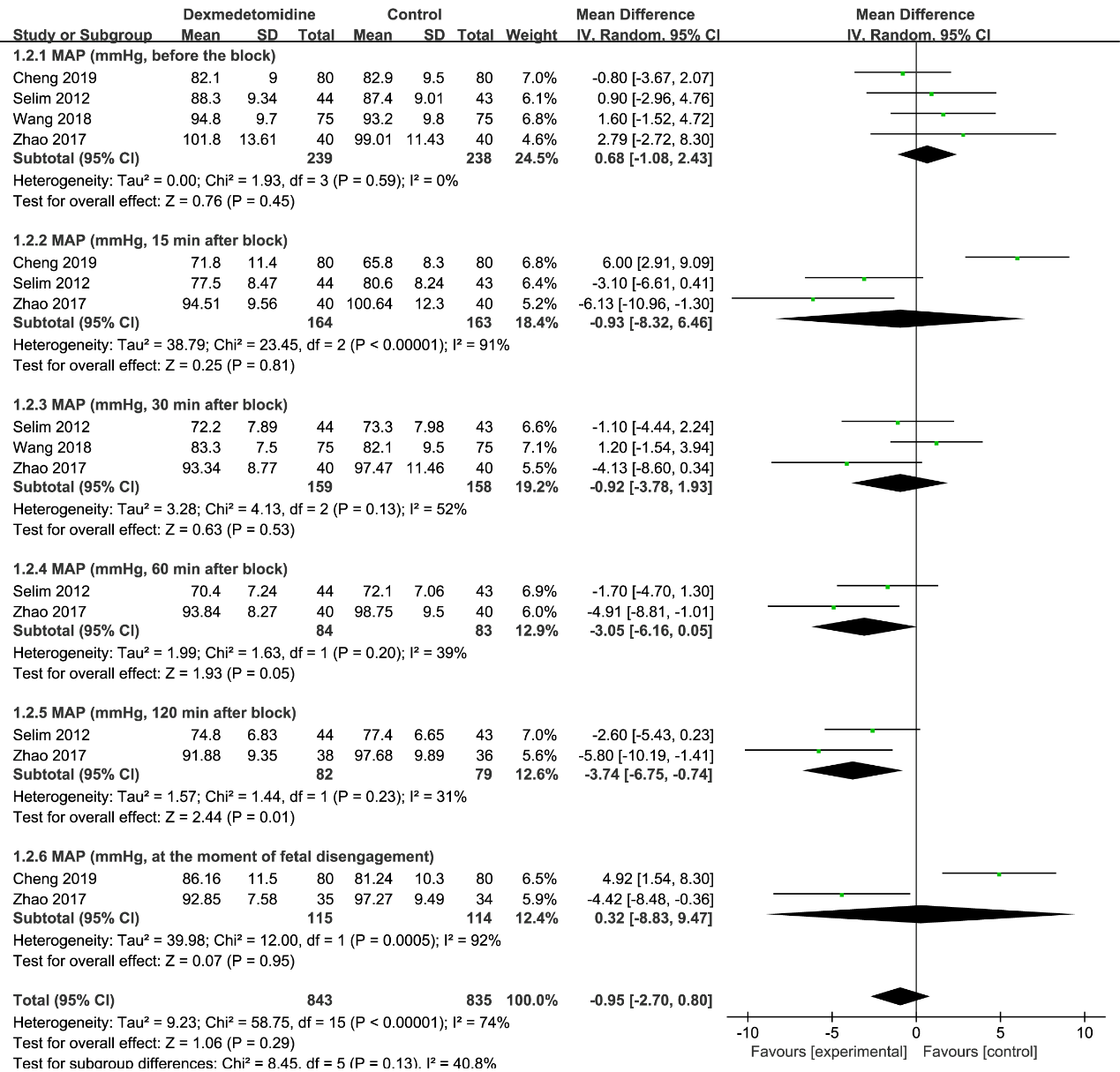


Supplementary figure. 2. Forest plot diagram showing the mean arterial pressure (MAP).


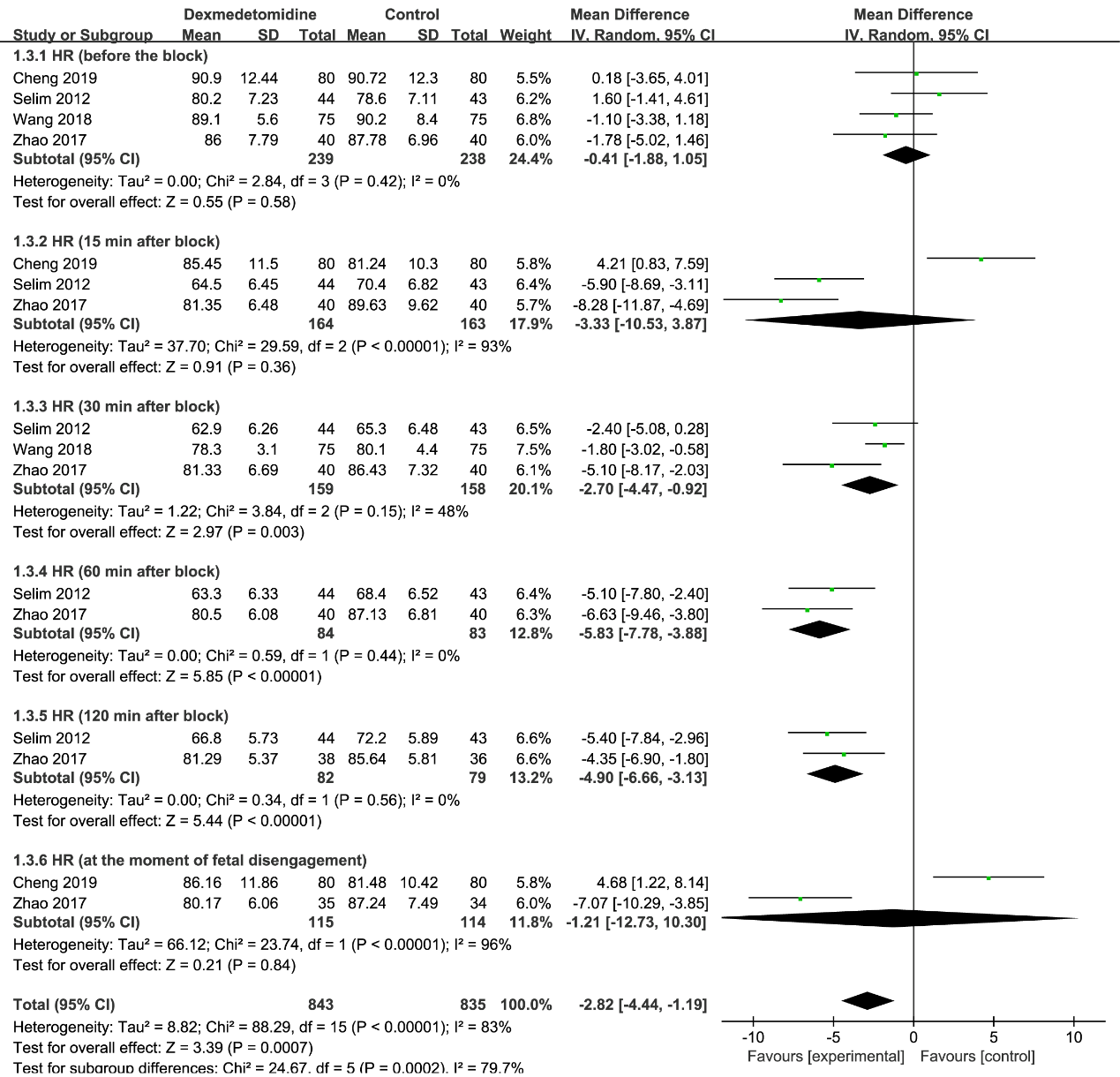


Supplementary figure. 3. Forest plot diagram showing the heart rate (HR).


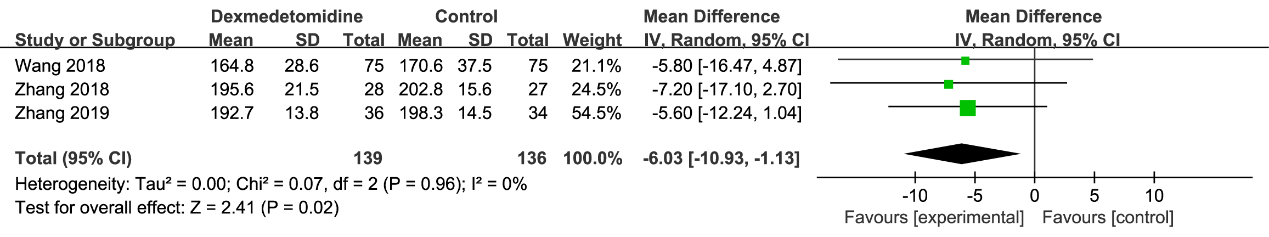


Supplementary figure. 4. Forest plot diagram showing the blood loss.


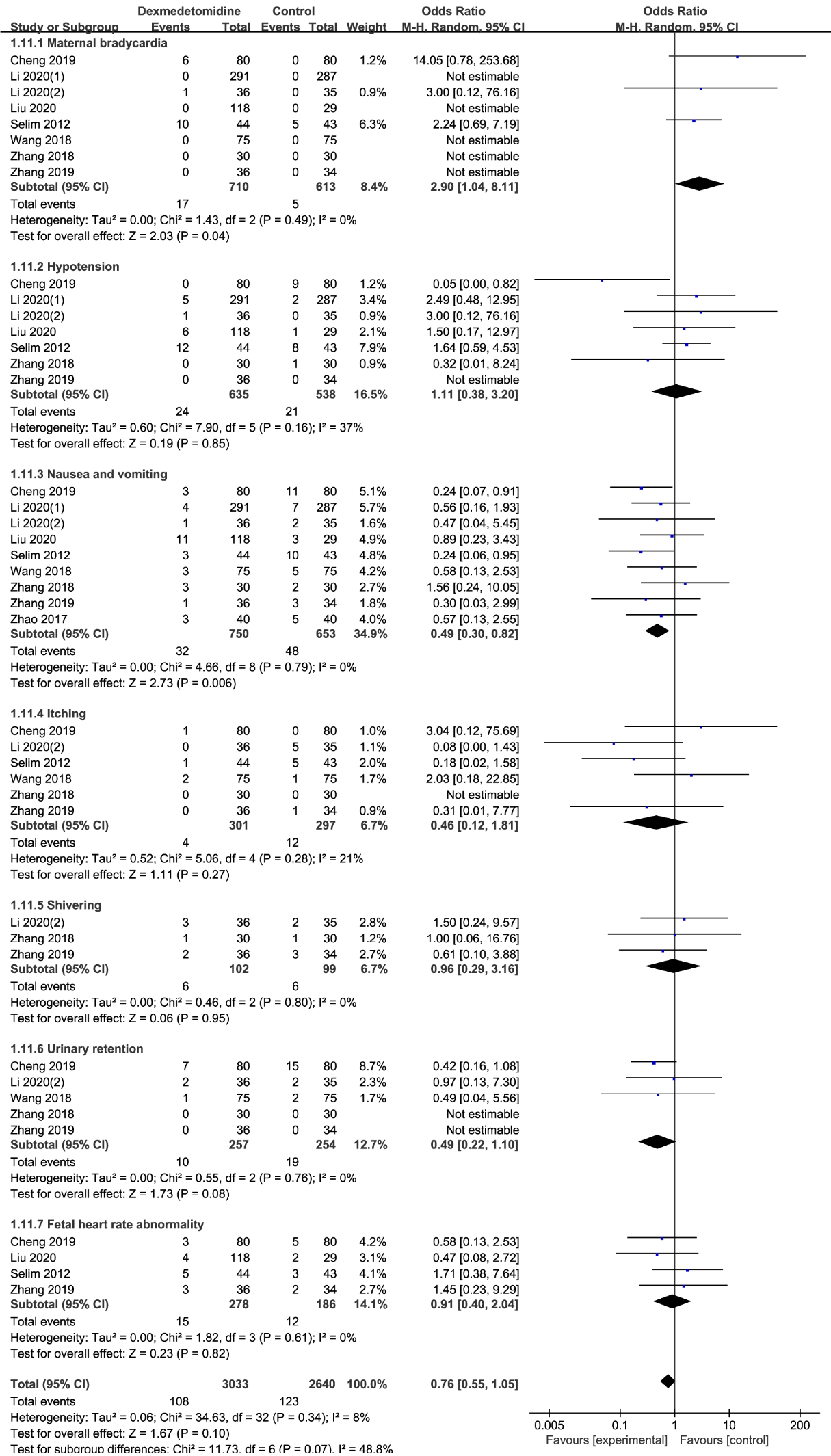


Supplementary figure. 5. Forest plot diagram showing the incidences of complications.


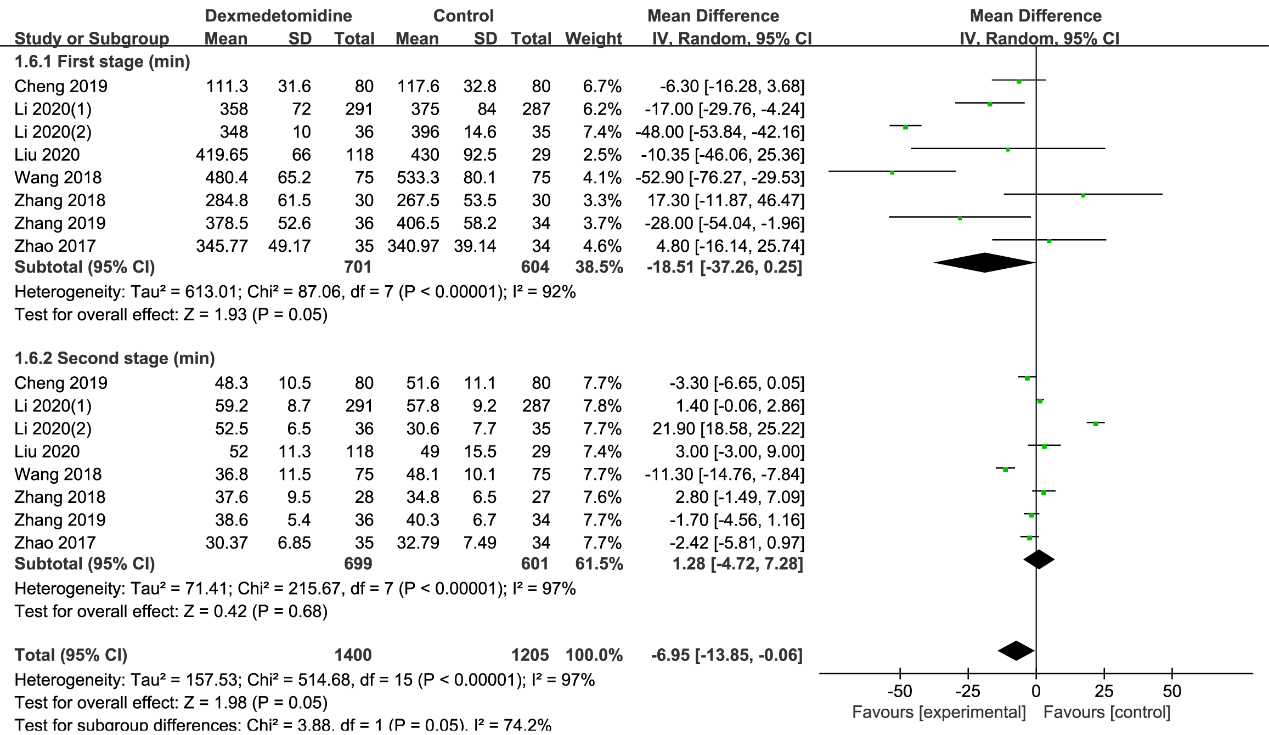


Supplementary figure. 6. Forest plot diagram showing the duration of labor stages.


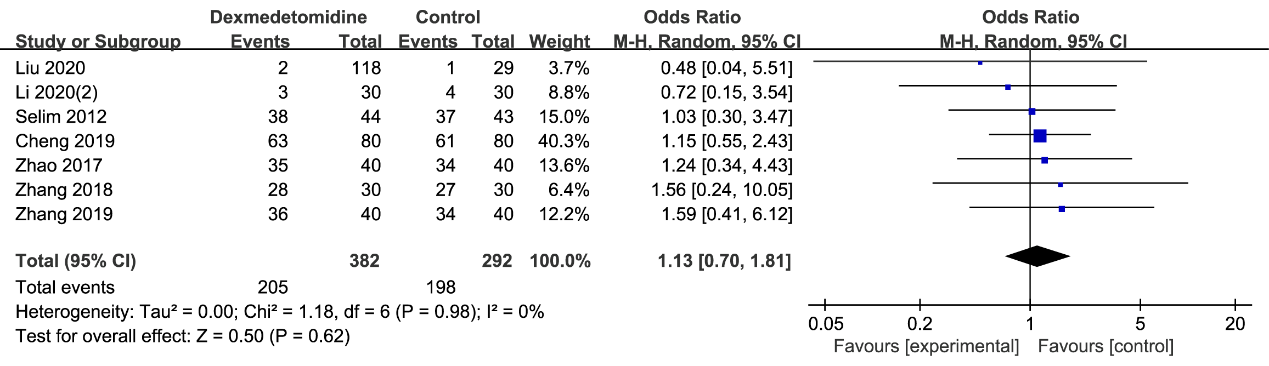


Supplementary figure. 7. Forest plot diagram showing the mode of delivery.


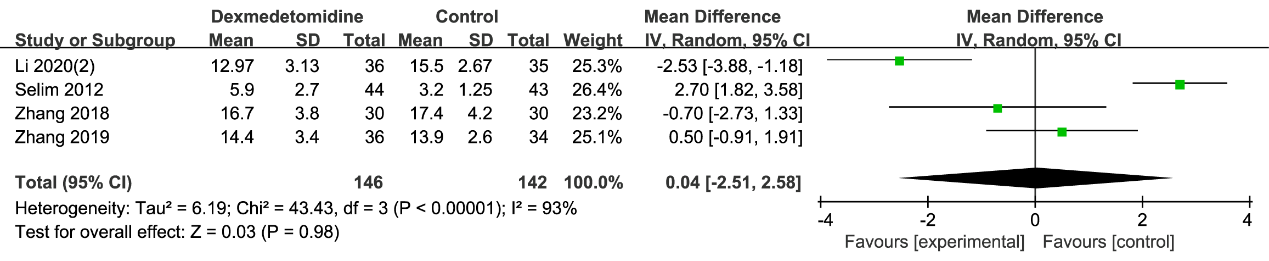


Supplementary figure. 8. Forest plot diagram showing the onset of analgesia.


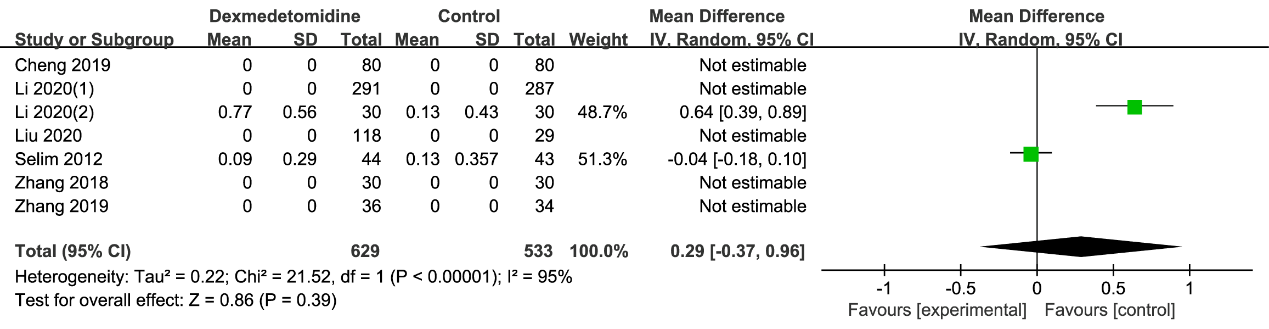


Supplementary figure. 9. Forest plot diagram showing the level of motor block.


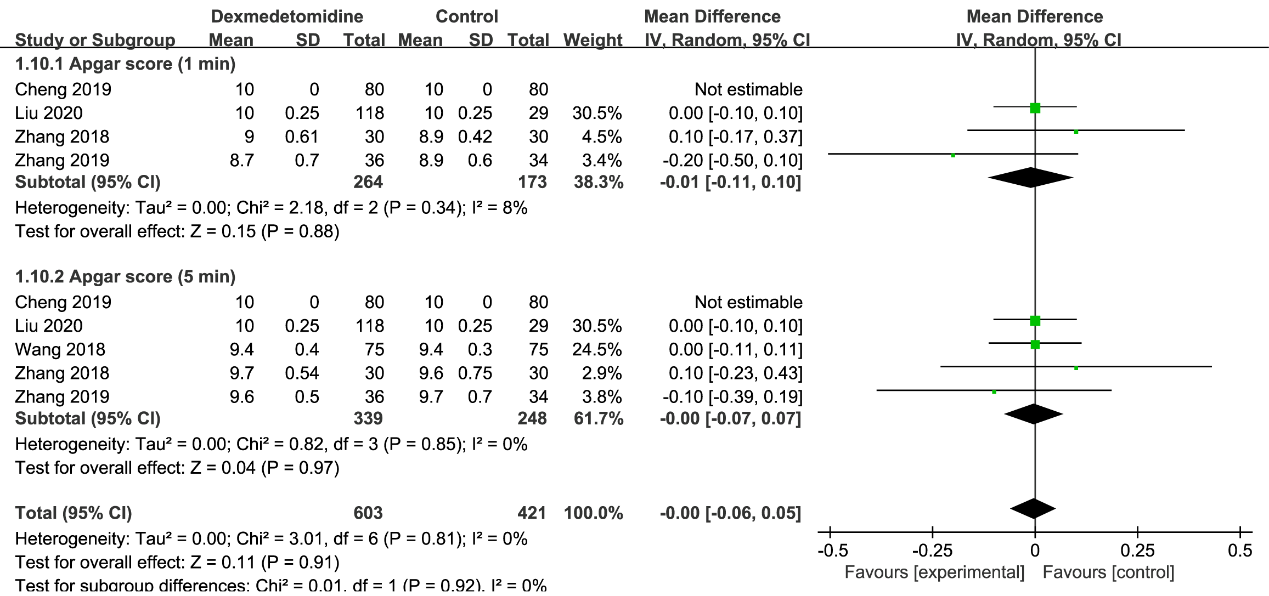


Supplementary figure. 10. Forest plot diagram showing the Apgar score.


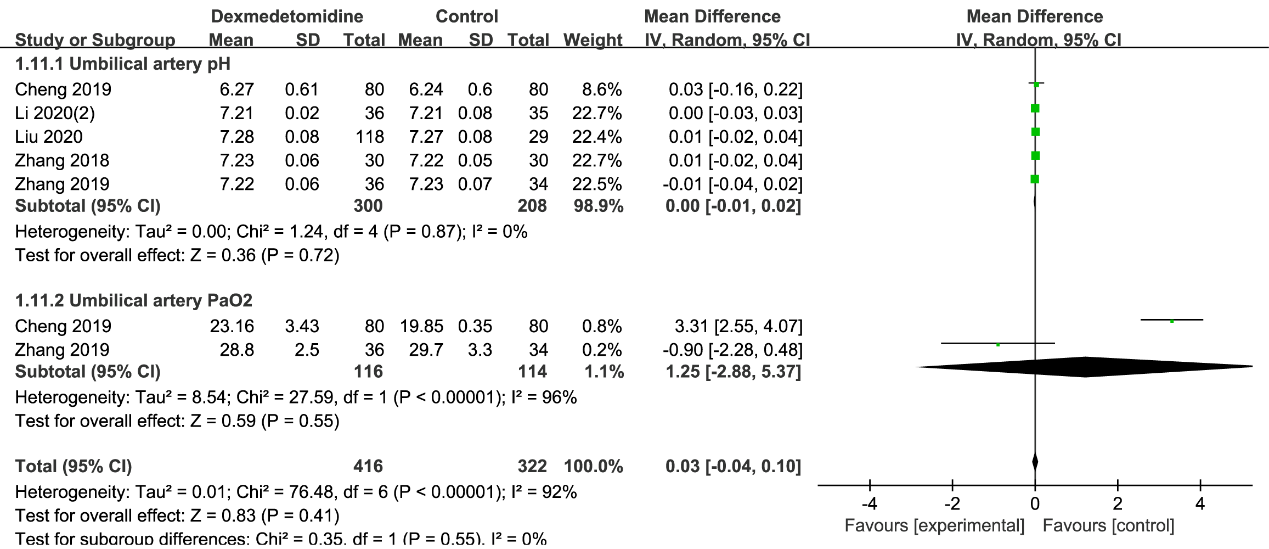


Supplementary figure. 11. Forest plot diagram showing the umbilical artery pH and PaO_2_.


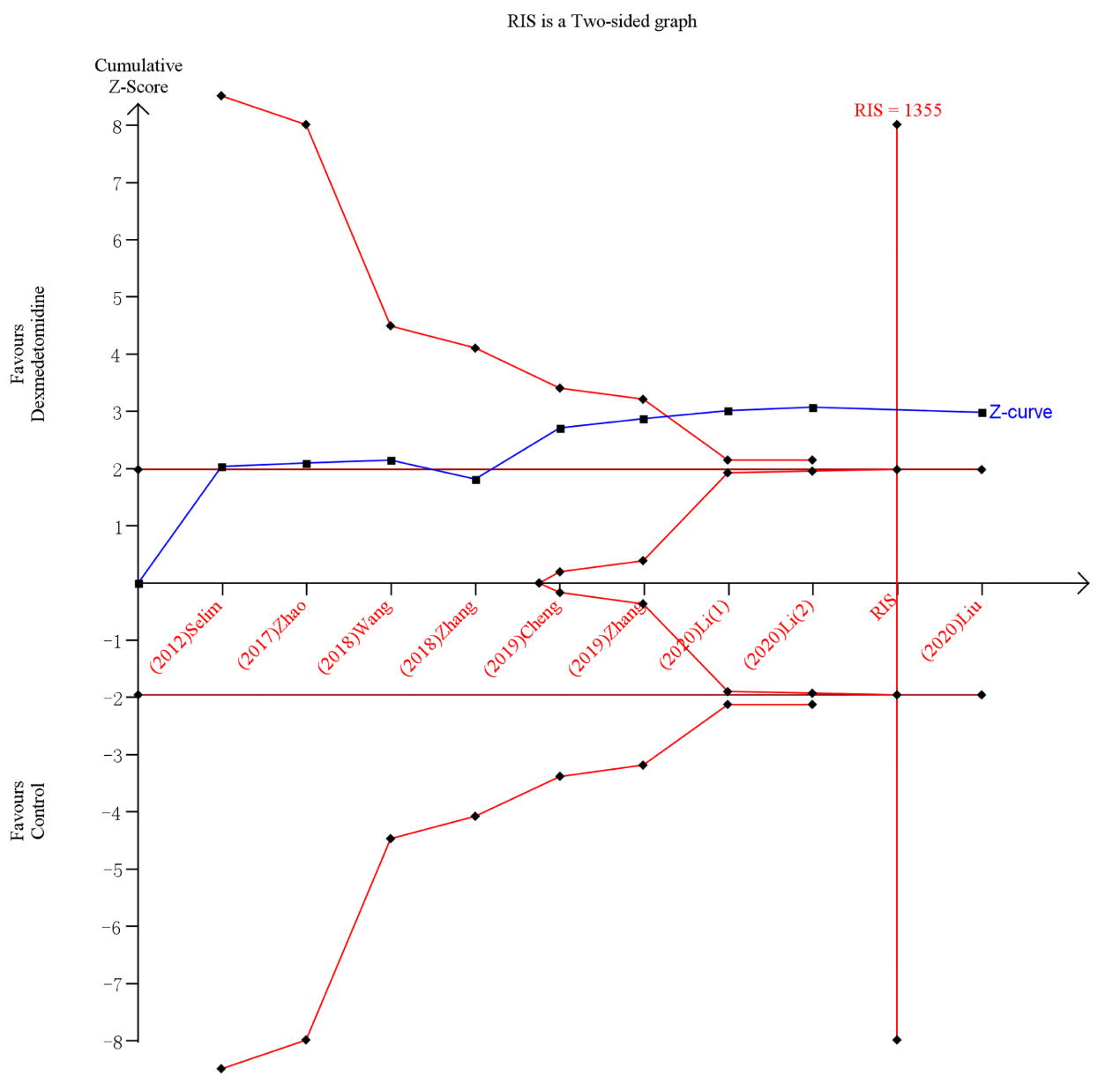


Supplementary figure. 12. Trial sequential analysis of the incidence of nausea/vomiting compared with placebo and opioids. RIS = required information size.
